# Proteomic analyses on chicken breast meat with white striping myopathy

**DOI:** 10.1016/j.psj.2024.103682

**Published:** 2024-03-22

**Authors:** Byungwhi Kong, Casey Owens, Walter Bottje, Majid Shakeri, Janghan Choi, Hong Zhuang, Brian Bowker

**Affiliations:** ⁎ USDA, Agricultural Research Service, U.S. National Poultry Research Center, Quality & Safety Assessment Research Unit, Athens, GA, USA; † Department of Poultry Science, Division of Agriculture, University of Arkansas System, Fayetteville, AR, USA

**Keywords:** broiler chicken, chicken breast myopathies, white striping, proteomics, postmortem

## Abstract

White striping (**WS**) is an emerging myopathy that results in significant economic losses as high as $1 billion (combined with losses derived from other breast myopathies including woody breast and spaghetti meat) to the global poultry industry. White striping is detected as the occurrence of white lines on raw poultry meat. The exact etiologies for WS are still unclear. Proteomic analyses of co-expressed WS and woody breast phenotypes previously demonstrated dysfunctions in carbohydrate metabolism, protein synthesis, and calcium buffering capabilities in muscle cells. In this study, we conducted shotgun proteomics on chicken breast fillets exhibiting only WS that were collected at approximately 6 h postmortem. After determining WS severity, protein extractions were conducted from severe WS meat with no woody breast (**WB**) condition (n = 5) and normal non-affected (no WS) control meat (n = 5). Shotgun proteomics was conducted by Orbitrap Lumos, tandem mass tag (**TMT**) analysis. As results, 148 differentially abundant proteins (|fold change|>1.4; *p*-value < 0.05) were identified in the WS meats compared with controls. The significant canonical pathways included BAG2 signaling pathway, glycogen degradation II, isoleucine degradation I, aldosterone signaling in epithelial cells, and valine degradation I. The potential upstream regulators include LIPE, UCP1, ATP5IF1, and DMD. The results of this study provide additional insights into the cellular mechanisms on the WS myopathy and meat quality.

## INTRODUCTION

Chicken breast myopathies causing quality defects have negatively impacted to world poultry production, and they were collectively estimated to cost the global poultry industry over $1 billion in both direct and indirect expenses (Barbut, 2020). White striping (**WS**) is a condition where fat is deposited along the muscle fibers of the breast muscles, mainly from cranially to caudally, in the direction of the myofibers (Kuttappan et al., 2013). Compared to normal breast meat, WS meat has higher lipid and collagen contents, and lower protein content (Petracci et al., 2014). The condition has been known to be linked to vascular inflammation and macrophage infiltration possibly due to the fast growth of the pectoralis major muscle, which disrupts its metabolism and homeostasis (Vanhatalo et al., 2021). Hypoxia, or low oxygen levels, is also believed to be involved in the formation of white striations. In hypoxic conditions, ATP production is reduced in the cells due to lack of oxygen for cellular respiration, which leads to disturbed ion gradients and vacuole formation (Boerboom et al., 2018).

To understand the etiology, genetic susceptibility, gene and protein expression, and biochemical pathways associated with breast myopathies, including WS and WB meat, researchers have extensively used genomics (Alnahhas et al., 2016; Pampouille et al., 2018), transcriptomics (Mutryn et al., 2015; Marchesi et al., 2019; Maharjan et al., 2021), proteomics (Kuttappan et al., 2017; Cai et al., 2018; Zhang et al., 2020a; Zhang et al., 2020b), metabolomics (Abasht et al., 2016; Boerboom et al., 2018; Consolo et al., 2020), and other methodologies (Abasht et al., 2021; Bordini et al., 2022; Bordini et al., 2021; Kong et al., 2021). Transcriptomics with WS meat identified differentially expressed genes associated with hypoxia, cell death, angiogenesis, immune system, and striated muscle contraction (Marchesi et al., 2019). Weighted gene co-expression network analysis (**WGCNA**) based on gene expression profiles obtained with WB and WS muscles suggested that alterations in extracellular matrix composition could activate the growth-related myopathies onset, and that collagen IV alterations may induce the endoplasmic reticulum stress response (Bordini et al., 2021). Proteomics studies with severe WB and WS muscles identified differentially expressed proteins associated with increased protein synthesis, decreased carbohydrate metabolic activity, decreased mitochondrial biogenesis, and reduced function of rapamycin independent companion of mammalian target of rapamycin (**RICTOR**) (Bottje et al., 2021; Kuttappan et al., 2017). However, global protein expression studies on WS-only muscle phenotype without other breast myopathy were limited in contrast to a variety of omics studies on WB (and WB with WS).

Shotgun proteomics based on high-resolution mass spectrometry (**MS**) facilitates the quantification of thousands of proteins, including their modifications, localization, turnover, and interaction partners (Altelaar et al., 2013; Mann et al., 2013). Unlike targeted approaches, shotgun proteomics provides a hypothesis-free analysis that complements antibody-based techniques. Advancements in MS-based proteomics including sample preparation, liquid chromatography (**LC**)-MS, and computational analysis, have establish shotgun proteomics as the preferred tool for characterizing protein dynamics across various functional dimensions. Therefore, proteomics relying on LC-MS has replaced traditional methods such as 2-dimensional gel electrophoresis (**2DGE**). Shotgun proteomics has been successfully employed in poultry muscle research (Kong et al., 2016; Kuttappan et al., 2017; Ouyang et al., 2017; Assuncao et al., 2023).

In this study, shotgun proteomics using liquid chromatography-tandem mass spectrometry (**LC-MS/MS**) was conducted with chicken breast fillets collected approximately 6 h postmortem that exhibited only the WS condition. Differentially abundant proteins in WS meat were identified compared to normal meat, and biological functions of differentially abundant proteins that affect meat quality were also determined.

## MATERIALS AND METHODS

### Ethics Statement

The care and experimental use of animal protocols were approved by the University of Arkansas Institutional Animal Care and Use Committee (**IACUC**#: 20016).

### Animal Husbandry

The animals were maintained according to a standard management program at the Poultry Farm, University of Arkansas. The general procedures of husbandry, feeding, handling, and slaughtering process were followed by Maynard (2023) with minor modifications. Briefly, 25 Cobb 700 broiler chicks were grouped and placed a floor pen (1.2 × 1.82 m; 0.09 m^2^ per bird). Experimental pens were furnished with fresh pine shavings, a hanging feeder, and a nipple drinker water line. Birds were allowed unrestricted access to feed and water. Internal house conditions were held constant with a set point temperature of 32°C when placed. Environmental temperature was reduced 2°C weekly until an endpoint temperature of 15°C was met. Lighting was maintained as hours of light (L) to hours of dark (D) as follows: 24L:0D from d 0 to 1, 23L:1D from d 1 to 7, and 16L:8D from d 7 to 56. Starter diets were fed as crumbles from d 0 to d 14, whereas the grower, finisher, and withdrawal diets were fed as pellets from 15 to 28, 29 to 42, and 43 to 56 d of age, respectively. At d 56, birds were transported in coops (Kuhl Coop Model 13-A, KUHL Corporation, Flemington, NJ), from chicken house to the University of Arkansas pilot processing plant (Fayetteville, AR). Following a 10 h feed withdrawal period, birds were hung on an inline shackle system, and processed with procedures by electrically stunned (11 V, and 11 mA for 11 s), exsanguinated, scalded in hot water (53.8°C, 2 min), and then defeathered. Prior to mechanical evisceration, necks and hocks were manually removed from each bird. Carcasses were moved to a 2-stage chilling system consisting of a 0.25 h prechill, at 12°C, before being placed in immersion chilling tanks held at 0°C for 2.5 h with manual agitation. At 3-h postmortem, carcasses were deboned, and breast meats were then utilized for further analysis (Maynard, 2023).

### Breast Muscle Sample Collection

Breast fillets without any distinct white lines were classified as normal (Con). Fillets that exhibited white lines, parallel to the muscle fibers, which were > 1 mm thick and very visible on the fillet surface were classified as severe white striping (**WS**) (Kuttappan et al., 2012). Five samples from different birds representing the Con category and 5 others representing the WS category were selected for proteomic analysis. The sampling locations of breast fillets were right pectoralis major muscle, cranial (proximal end)-superficial (5 mm deep under the skin) area (Sun et al., 2022), and sample dimensions were approximately 10 mm × 10 mm × 10 mm. Samples were collected at approximately 6 h postmortem, snap-frozen, and stored at −80°C until processing for proteomic analysis.

### Muscle Extracts

Extractions from chicken breast meat were conducted following procedures by Kong et al. (2016) and Kuttappan et al. (2017) with modifications. Muscle samples (0.5 g) were homogenized in 1.5 mL of RIPA lysis buffer (25 mM Tris-HCl pH 7.6, 150 mM NaCl, 1%, NP-40, 1% sodium deoxycholate, 0.1% SDS; ThermoFisher Scientific, Waltham, MA) for total protein extraction using a hand-held Tissue-Tearor (Biospec Products Inc., Bartlesville, OK) at speeds varying from 5,000 rpm to 32,000 rpm. Following homogenization, samples were centrifuged at 10,000 × *g*, and the supernatant was collected. Protein concentrations of the supernatants were determined by the Bradford method using the Bio-Rad protein assay kit (Bio-Rad, Hercules, CA). Extractions were then diluted to a protein concentration of 20 μg /150 μL using 1x phosphate buffered saline (PBS), and the samples were stored at −80°C until further analysis.

### Shotgun Proteomics and Statistical Analysis

Shotgun proteomic and statistical analysis followed procedures by Kong et al. (2016) and Kuttappan et al. (2017) with modifications. Briefly, protein samples were subjected to shotgun proteomics analysis by trypsin digestion and tandem mass spectrometry (**MS/MS**) at the University of Arkansas Medical Science (**UAMS**) Proteomics Core Facility (Little Rock, AR). Raw mass spectrometric data were analyzed by database searching using the Mascot (Matrix Science, Boston, MA) search engine and the UniProtKB database (http://www.uniprot.org/help/uniprotkb). Search results were compiled using Scaffold program (Proteome Software, Portland, OR). Raw spectral counts were transformed to log_2_ values and normalized by Loess method using JMP Genomics (SAS Institute, Cary, NC). Differential abundances between Con and WS were calculated using log_2_fold change (**FC**), indicating |log_2_FC of 0.44| = |FC of 1.4| in numeric value. The t-test was used for proteomics data in comparing Con and WS samples. Proteins showing *p* < 0.05 in the comparison between Con and WS were considered differentially abundant. The *p*-value correction (FDR calculation) by multiple tests was not applied in this study since we used a less stringent approach on a molecule by molecule basis, which allowed us to import more informative lists. Heatmapping for hierarchical clustering analysis was conducted with the online Heatmapper analytical tool using the average linkage method and Euclidean distance measurement method at http://www.heatmapper.ca/expression/ (Babicki et al., 2016).

### Pathway Analysis

Ingenuity Pathway Analysis (IPA; Qiagen, Valencia, CA; http://www.ingenuity.com) software was used to perform functional annotation, canonical pathway, upstream analyses and network discovery (Kong et al., 2016; Kong et al., 2017; Kuttappan et al., 2017; Khatri et al., 2018). Since IPA is based on the bioinformatics in humans, functionalities for differentially abundant proteins in our chicken datasets are based primarily on mammalian biological mechanisms. As the investment in biomedical research biases the functional annotations towards human disease, we have attempted to draw plausible conclusions based on avian based literature.

## RESULTS AND DISCUSSION

### Shotgun Proteomics Results

**Figure 1:**
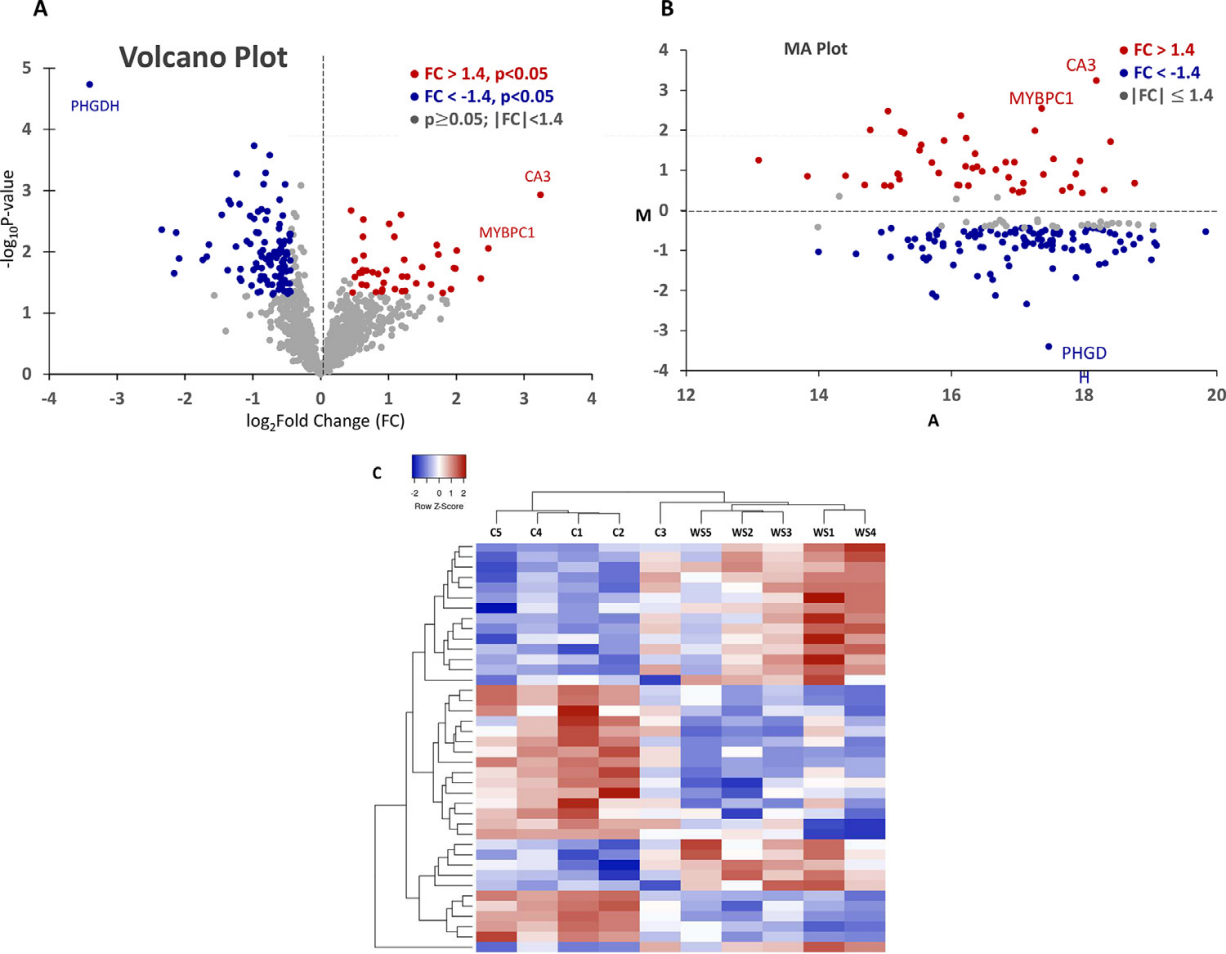
Overview of proteomics. (A) Volcano plot for all 1051 proteins identified by shot-gun proteomics. Legends for colored dots displayed inside of the plot (FC, fold change; p, p value). Representative protein names were indicated. (B) MA plot for proteins showing p<0.05. M means log_2_FC in y-axis and A means average log_2_ spectral intensity values between normal and WS meat samples. (C) Heatmap for hierarchical clustering analysis. Red and blue colors indicate higher- and lower abundances, respectively.

The global protein distribution of differential expression identified 1051 proteins (Additional file 1) and log_2_fold change (log_2_FC) and -log_10_P-value were plotted as a volcano plot in Figure 1A. Among those, differential abundance levels for 195 proteins were statistically significant (*p*-value < 0.05) and 148 differentially abundant proteins including 43 higher- and 105 lower abundances with |log_2_FC|>0.44 (∼1.4-fold difference) were identified in the WS meat compared with normal meat as shown in MA plot (Figure 1B). Of those, the top 20 higher- and 20 lower abundant proteins in WS meat were subjected to hierarchical clustering analysis (Figure 1C), resulting in the correct group of origin for all 10 birds being clearly discriminated, although one control sample (Con3) was slightly biased to WS meat sample. A list of the 10 most up- and downregulated proteins (Table 1) in WS meat were presented. Of those, increased abundance of vimentin (**VIM**), which is a muscle-specific protein maintaining muscle cytoarchitecture (along with desmin, though not found in differentially abundant protein list in this study), has been known as a marker of breast myopathies including WS, WB, and spaghetti meat (Soglia et al., 2019).

**Table 1:** Top 10 up- and down-regulated proteins.

Symbol	Entrez gene name	Log2FC	*p-value*
Higher in WS			
CA3	carbonic anhydrase 3	3.2	0.00117
MYBPC1	myosin binding protein C1	2.5	0.00877
FHL1	four and a half LIM domains 1	2.4	0.02730
TRIM54	tripartite motif containing 54	2.0	0.00953
AMPD1	adenosine monophosphate deaminase 1	2.0	0.01880
DUSP29	dual specificity phosphatase 29	2.0	0.01840
VIM	vimentin	1.9	0.04080
MAPK14	mitogen-activated protein kinase 14	1.8	0.04700
CAMK2D	calcium/calmodulin dependent protein kinase II delta	1.7	0.01110
APOA1	apolipoprotein A1	1.7	0.00773
Lower in WS			
PHGDH	phosphoglycerate dehydrogenase	−3.4	0.00002
COL1A1	collagen type I alpha 1 chain	−2.3	0.00435
PSAT1	phosphoserine aminotransferase 1	−2.2	0.02250
COL1A2	collagen type I alpha 2 chain	−2.1	0.00484
ASNS	asparagine synthetase (glutamine-hydrolyzing)	−2.1	0.01290
MUSTN1	musculoskeletal, embryonic nuclear protein 1	−1.7	0.01360
MYBPH	myosin binding protein H	−1.7	0.01200
IL18	interleukin 18	−1.6	0.00762
EDF1	endothelial differentiation related factor 1	−1.5	0.00249
TXLNB	taxilin beta	−1.4	0.01990

Lower abundances of collagen type 1 isoforms (**COL1A1** and **COL1A2**) were observed in WS meat, which contrasted with increased general collagen contents observed in WS meat in earlier studies (Petracci et al., 2014; Carvalho et al., 2021). In addition, Carvalho et al., (2021) only reported sarcoplasmic extracts in severe WS meat, but not in normal meat, detected collagen type 6 alpha 3 chain (**COL6A3**) together with higher levels of other muscle specific proteins (e.g., troponin subtypes). Collectively, the earlier- and current findings indicate that collagen type 1 chains may not be involved in the increased general collagen content caused by overall increased connective tissue found in WS meat. Further studies may be needed to investigate composition of biochemical components in increased connective tissues found in chicken breast myopathies and understand the causes and effects of increased connective tissues. The higher abundance of myosin binding protein C1 (**MYBPC1**), which represents slow twitch muscle (dark meat), and the lower abundance of myosin binding protein H (**MYBPH**), which represents fast twitch muscle (white meat), suggests muscle type fiber switching in WS samples, which was earlier found in breast myopathies (Lake et al., 2021).

### Bioinformatic Pathway Analysis

**Figure 2:**
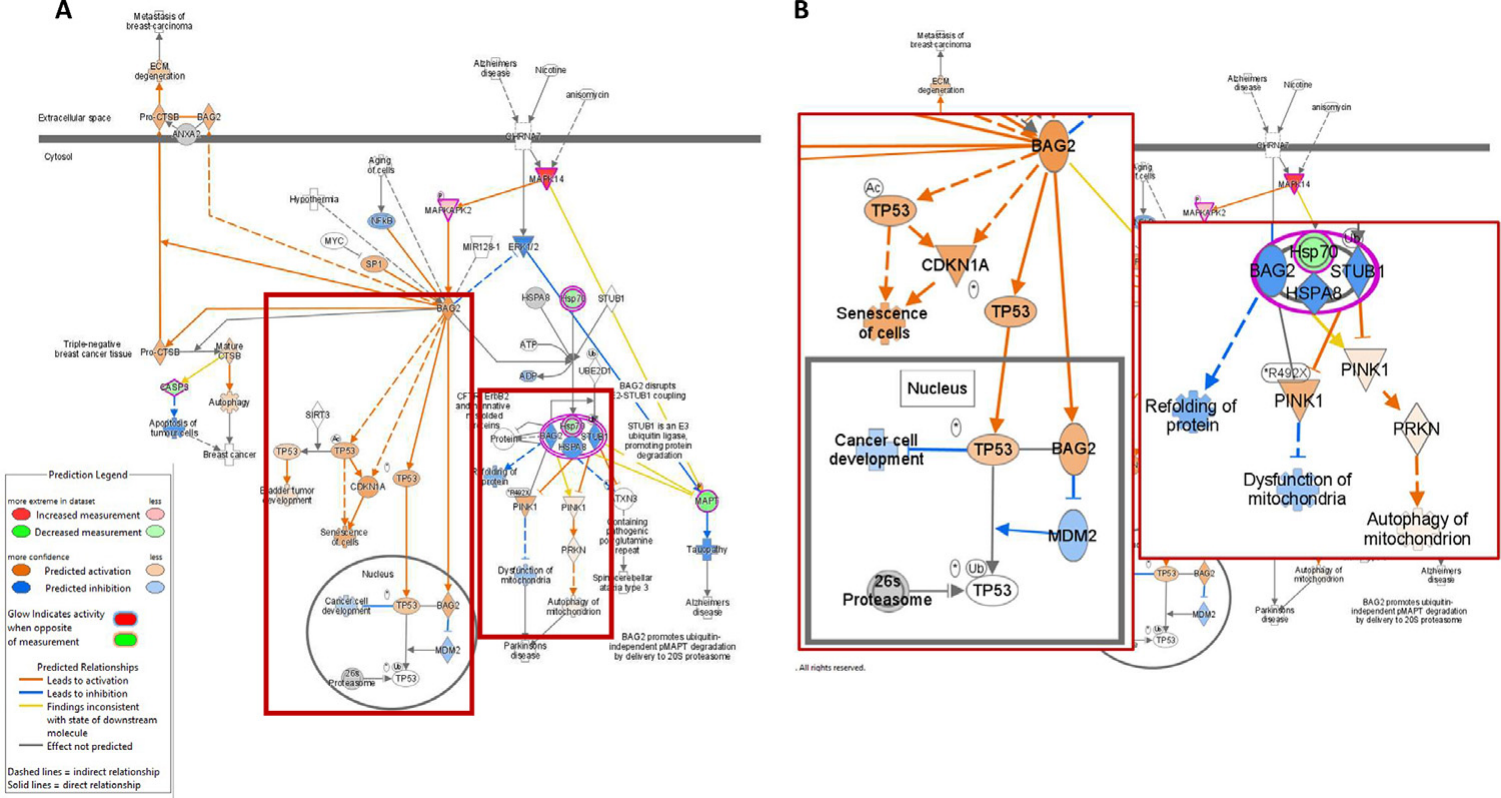
BAG2 signaling pathway. (A) overall pathway related to BAG2 and HSP70. (B) Enlarged image of red boxes in Figure 2A. Color legends for shapes, lines, and arrows used in the figure were displayed in the left side of Figure 2A.

The functional annotation and biological pathway analyses for 148 differentially abundant proteins were analyzed by IPA. From this analysis, 362 canonical pathways were generated (data not shown) and the 10 most significant pathways with *p*-value and involved molecules were listed in Table 2. Of those pathways, Bcl2-associated athanogene (**BAG2**) signaling pathway is known to interact with heat shock proteins (e.g., **HSP70** and **HSPA8**) and function in molecular co-chaperone (Qin et al., 2016). This pathway may interact with the tumor protein 53 (**p53**) pathway and play a role in cell death and organismal injury/abnormalities (Figure 2A) (Yue et al., 2015). In addition, the BAG2 signaling pathway appears to be involved in muscle degeneration through protein refolding, mitochondrial dysfunction, and mitochondrial autophagy (termed as mitophagy, Figure 2B) (Bueno et al., 2015). The BAG signaling pathway had not been found in the earlier proteomics study with WS and WB co-affected meat samples (Kuttappan et al., 2017). Thus, this BAG signaling pathway could be an additional mechanism to induce WS in chicken breast meat.

**Table 2:** Top 10 canonical pathways.

Ingenuity canonical pathways	*-log(p-value)*	zScore	Molecules	Physiological mechanisms in WS meat
BAG2 signaling pathway	3.93		CASP3, HSPA4, HSPA9, MAPK14, MAPKAPK2, MAPT	Co-chaperone with HSP70/80 & necrosis (Qin et al., 2016)
Glycogen degradation III	3.85		GAA, MTAP, PGM5	Decreased carbohydrate metabolisms (Kuttappan et al., 2017)
Isoleucine degradation I	3.34		ACAA2, BCAT1, DLD	Decreased branched chain aa catabolism (Boerboom et al., 2018; Holecek, 2018)
Aldosterone signaling in epithelial cells	3.20		CRYAB, DNAJB4, HSPA4, HSPA9, HSPD1, HSPE1, HSPH1	Blood pressure control/ hypoxia (Ayansola et al., 2021; Xanthakis and Vasan, 2013)
Valine degradation I	3.11		BCAT1, DLD, HIBADH	Decreased branched chain aa catabolism (Holecek, 2018)
Protein ubiquitination pathway	3.00		CRYAB, DNAJB4, HSPA4, HSPA9, HSPD1, HSPE1, HSPH1, THOP1, UBR1	Protein degradation (Boerboom et al., 2018)
HIF1α signaling	2.94	1.9	CAMK2D, CAMK2G, EIF4E, EIF4EBP1, HSPA4, HSPA9, VIM	Hypoxia (Malila et al., 2019)
Mitochondrial dysfunction	2.53	0.3	CAMK2D, CAMK2G, CASP3, CLIC2, COX17, DLD, GPX4, MAPT, SOD1	Mitochondrial Dysfunction (Prisco et al., 2021)
Glycogen degradation II	2.40		MTAP, PGM5	Decreased carbohydrate metabolism (Kuttappan et al., 2017)
Natural killer cell signaling	2.36		COL1A1, COL1A2, HSPA4, HSPA9, IL18, MAPK14	Immune cell infiltration (Prisco et al., 2021)

Table 2 also listed affected physiological mechanisms possibly linked to the canonical pathway. As shown in references in the table, canonical pathways appeared to be consistent with WS phenotypic/ biochemical characteristics reflected by altered cellular mechanisms (e.g., carbohydrate metabolism, protein degradation, hypoxia, mitochondrial dysfunctions) and histological findings (e.g., necrosis and immune cell infiltrations) identified from previous reports of chicken breast myopathies. Thus, canonical pathway analysis confirmed that WS meat showed similar mechanistic alterations in physiological systems that induce chicken breast myopathies.

### Upstream Regulators

**Figure 3:**
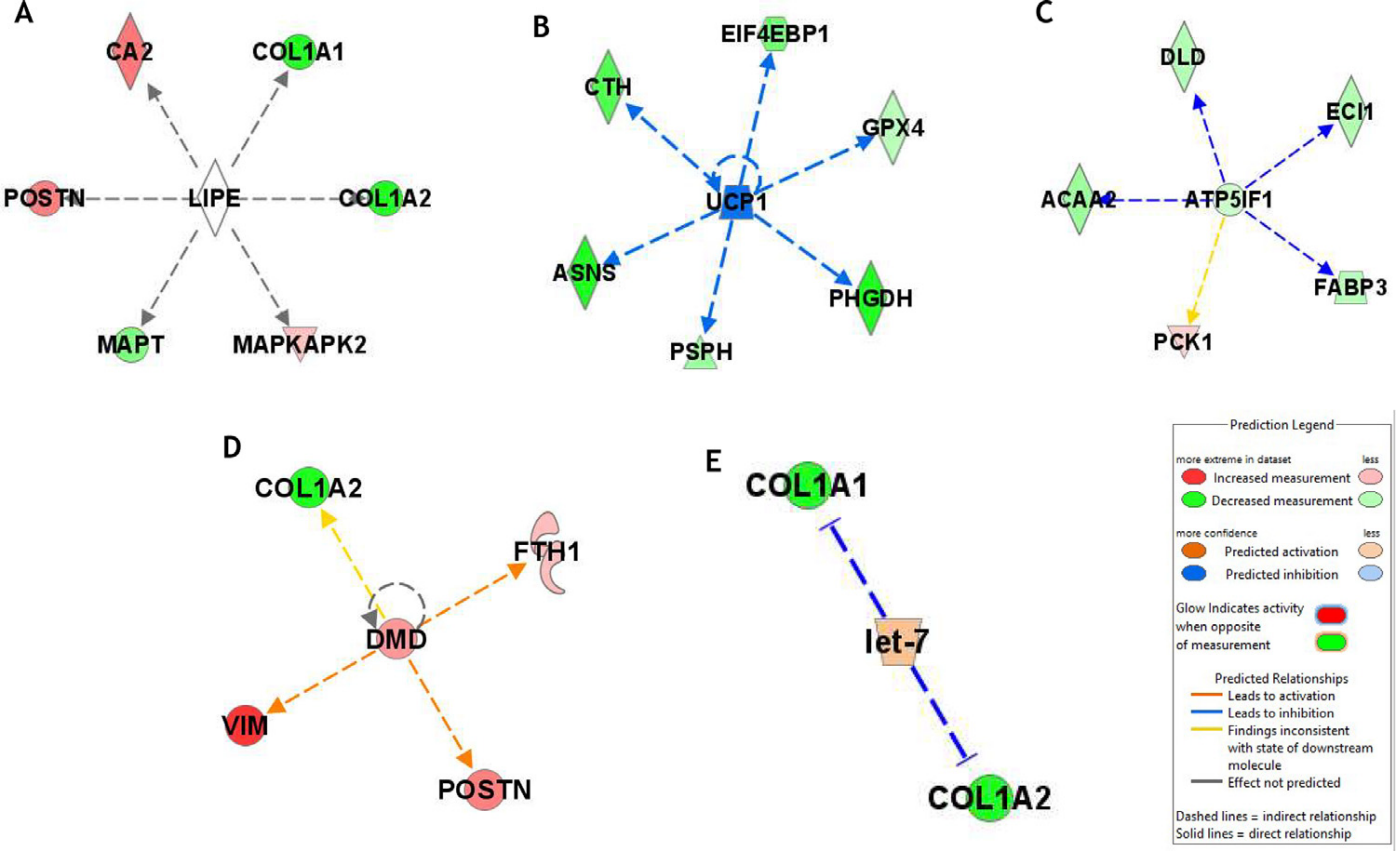
Molecular interactions centered with potential regulators. Legends for shapes, lines, and arrows were displayed in the right, bottom corner in Figure 3.

Table 3 listed potential upstream regulators for the phenotypic expression of WS meat and molecular interactions were shown in Figure 3. Of those, potential involvement of lipase E (**LIPE**) (Figure 3A) was previously reported as a regulator of differential muscle phenotypes for myopathy incidence and types of breeds (Kong et al., 2017; Papah and Abasht, 2019). Likewise, downregulated uncoupling protein 1 (**UCP1**) (UCP3 as chicken homolog; Figure 3B; activation z score = -1.94) was identified in the previous report (Kuttappan et al., 2017), indicating mitochondrial dysfunctions. ATP Synthase 5 Inhibitory Factor Subunit 1 (**ATP5IF1**; Figure 3C) appeared to decrease more rapidly in WS meat along with lower abundances of downstream effector proteins including fatty acid binding protein 3 (**FABP3**) as an indicator of decreased carbohydrate metabolism (Kuttappan et al., 2017). Dystrophin (**DMD**; Figure 3D), which is a major muscle protein, showed higher abundance in WS meat associated with lower collagen type 1 alpha 2 chain (**COL1A2**) and higher VIM (Rouger et al., 2002), thus, higher abundance of DMD may affect texture related meat quality postmortem. Likewise, let-7 microRNA (Figure 3E) may be upregulated due to the possible regulation of decreased abundance of collagen 1 isoforms (Mohamed et al., 2015). Proteins that may potentially regulate meat quality will be discussed elsewhere in this paper.

**Table 3:** Upstream regulators for causal networks.

Upstream regulator	Log2FC	Activation z-score	*p-value*	Target molecules
LIPE			0.000121	CA2, COL1A1, COL1A2, MAPKAPK2, MAPT, POSTN
UCP1		1.941	0.000153	ASNS, CTH, EIF4EBP1, GPX4, PCK1, PHGDH, PSPH
ATP5IF1	0.402		0.0002	ACAA2, DLD, ECI1, FABP3, PCK1
DMD	0.34		0.000345	COL1A2, FTH1,POSTN, VIM
let-7			0.00318	COL1A1, COL1A2

Previously, a shotgun proteomics study, which was conducted with WS and WB co-affected muscles (**WS/WB**) collected at ∼5 min postmortem compared with normal muscles, showed potentially altered physiological mechanisms, including carbohydrate metabolism, protein synthesis, oxidative stress and mitochondrial dysfunctions (Kuttappan et al., 2017; Bottje et al., 2021). When compared differentially abundant proteins of WS only muscle collected at 6 h postmortem (WS-6h) in this study with those in WS/WB muscles collected at ∼5 min postmortem (WS/WB-5min) in the previous study, proteins showing the same differential trends may reflect similar protein stabilities between WS and normal meat during processing procedures and short-term storage. The list of differentially abundant proteins in WS-6h included potential etiological factors such as carbonic anhydrase 3 (**CA3**), VIM, FABP3, reflecting protein carbonylation, skeletal muscle abnormalities, and decreased carbohydrate metabolisms. In contrast, proteins showing opposite differential trends between WS-6h and WS/WB-5 min may represent effector molecules to characterize meat quality. Proteins exhibiting greater abundance in WS/WB-5min, but lower in WS-6h include adenosine monophosphate deaminase 1 (**AMPD1**), dual specificity phosphatase 29 (**DUSP29**), calcium/calmodulin dependent protein kinase II delta (**CAMK2D**), crystallin alpha B (**CRYAB**), carbonic anhydrase 2 (**CA2**), that may indicate differential stability or rapid postmortem degradations in WS meat. AMPD1 is known to catalyze the deamination of AMP to IMP in skeletal muscle and plays an important role in the purine nucleotide cycle. Interestingly, a single nucleotide polymorphism (**SNP**) in the promoter region of the AMPD1 gene in chicken appears to modulate transcriptional activity of AMPD1 expression and may affect the freshness of chicken breast meat (Yu et al., 2021). Taken together, lower AMPD1 abundance may play a role in inducing breast myopathies, but the stabilities were higher affecting WS meat quality postmortem. CAMK2D is a protein that plays a role in calcium homeostasis. The increased mRNA expression of an alpha isoform (**CAMK2A**) of this protein in WS meat was reported previously (Marchesi et al., 2019), indicating calcium dyshomeostatic conditions observed in chicken breast myopathies including WS meat (Zhang et al., 2023). CRYAB is a member of the small heat shock protein family which functions as a molecular chaperone (Dimauro and Caporossi, 2022). Earlier studies showed increased mRNA expression of CRYAB in chicken breast myopathies (Zambonelli et al., 2016; Pampouille et al., 2019), which is consistent with higher abundance in WS meat in this study. CRYAB exhibits diverse functions in skeletal muscles including protecting muscle tissues from alterations of protein stabilities in structuring microfilaments, microtubules, and intermediate filament components (Dimauro and Caporossi, 2022). Therefore, greater abundance of CRYAB in WS meat may affect meat/texture quality in addition to being a marker of inducing chicken breast myopathies including WS. The CA2 (together with CA3) enzyme may play an important role in scavenging oxygen radicals and thereby protecting muscle cells from oxidative damage (Zimmerman et al., 2004), suggesting that the CA enzymes may be a marker of oxidative stress in chicken breast myopathies. Certain proteins were observed as numerically greater abundance in WS/WB-5min muscle, but significantly lower in WS-6h meat and they are mostly enzymes (superoxide dismutase 1, hydroxysteroid dehydrogenase like 2, acetyl-CoA acyltransferase 2, aldehyde dehydrogenase 7 family member A1). This result indicates more rapid degradation of enzymes in WS meat which may be due to higher levels of oxidized proteins found in WS breast meat (Costa Filho et al., 2023). Of those, superoxide dismutase 1 (**SOD1**) and thioredoxin were significantly less abundant in WS-6h meat, and this result is consistent with an earlier report of dramatic decreases in antioxidant activities in severe WS meat (Carvalho et al., 2021).

## SUMMARY AND CONCLUSIONS

This study has identified differentially abundant proteins in WS meat collected at 6 h postmortem compared to normal meat. Many of these proteins were consistent with previous proteomics results on chicken breast myopathies. Additionally, potentially important proteins were identified for etiological pathways (e.g., BAG2 signaling pathway, branched chain amino acid catabolism) and as potential influencers of meat quality development (e.g., fiber type switch with myosin binding protein, decreased collagen subtypes, COL1A1 and COL1A2, VIM, CRYAB). These results provide additional insights into protein factors that can mediate meat quality of chicken breast myopathies.

## Appendix Supplementary materials

Image, application 1
